# Influence of Abiotic and Biotic Elicitors on Organogenesis, Biomass Accumulation, and Production of Key Secondary Metabolites in Asteraceae Plants

**DOI:** 10.3390/ijms25084197

**Published:** 2024-04-10

**Authors:** Maria Petrova, Kamelia Miladinova-Georgieva, Maria Geneva

**Affiliations:** Institute of Plant Physiology and Genetics, Bulgarian Academy of Sciences, Acad. G. Bonchev Street, Bldg. 21, 1113 Sofia, Bulgaria; marry_petrova@yahoo.com (M.P.); kameca@abv.bg (K.M.-G.)

**Keywords:** in vitro culture, hairy roots, shoot culture, cell suspension, secondary metabolites, biosynthetic genes, elicitation

## Abstract

The medicinal plants of the Asteraceae family are a valuable source of bioactive secondary metabolites, including polyphenols, phenolic acids, flavonoids, acetylenes, sesquiterpene lactones, triterpenes, etc. Under stressful conditions, the plants develop these secondary substances to carry out physiological tasks in plant cells. Secondary Asteraceae metabolites that are of the greatest interest to consumers are artemisinin (an anti-malarial drug from *Artemisia annua* L.—sweet wormwood), steviol glycosides (an intense sweetener from *Stevia rebaudiana* Bert.—stevia), caffeic acid derivatives (with a broad spectrum of biological activities synthesized from *Echinacea purpurea* (L.) Moench—echinacea and *Cichorium intybus* L.—chicory), helenalin and dihydrohelenalin (anti-inflammatory drug from *Arnica montana* L.—mountain arnica), parthenolide (“medieval aspirin” from *Tanacetum parthenium* (L.) Sch.Bip.—feverfew), and silymarin (liver-protective medicine from *Silybum marianum* (L.) Gaertn.—milk thistle). The necessity to enhance secondary metabolite synthesis has arisen due to the widespread use of these metabolites in numerous industrial sectors. Elicitation is an effective strategy to enhance the production of secondary metabolites in in vitro cultures. Suitable technological platforms for the production of phytochemicals are cell suspension, shoots, and hairy root cultures. Numerous reports describe an enhanced accumulation of desired metabolites after the application of various abiotic and biotic elicitors. Elicitors induce transcriptional changes in biosynthetic genes, leading to the metabolic reprogramming of secondary metabolism and clarifying the mechanism of the synthesis of bioactive compounds. This review summarizes biotechnological investigations concerning the biosynthesis of medicinally essential metabolites in plants of the Asteraceae family after various elicitor treatments.

## 1. Medicinal Plants from Asteraceae Family: Chemical Constituents and Applications

The Asteraceae family is one of the largest families of flowering plants in the world, with over 1600 genera and about 32,000 species [1]. Most of the Asteraceae family members have been used in medicine for centuries because of their various therapeutic applications. The plant species of Asteraceae contain a wide range of biologically active compounds; major among them are phenolic acids, flavonoids, terpenoids, volatile components, acetylenes, etc. [2]. They exhibit strong antibacterial, anti-inflammatory, antioxidant, anticancer, and antiparasitic activities, as well as diuretic and wound-healing qualities [3]. Several well-known species, such as *Artemisia annua* (sweet wormwood), *Stevia rebaudiana* (stevia), *Echinacea purpurea* (echinacea), *Chicorium intybus* (chicory), *Arnica montana* (mountain tobacco), *Tanacetum parthenium* (feverfew), and *Silybum marianum* (milk thistle), are of great interest to scientists because of their multispectral healing effects and wide uses in medicine, functional food, and cosmetic products.

*Artemisia annua* L. (sweet wormwood) is a herbaceous medicinal plant, the only commercial and economical source of the sesquiterpene lactone artemisinin, efficiently applied in the treatment of cerebral malaria fever worldwide. Apart from its malarial properties, Artemisia spp. has been studied for its wide range of biological activities, including metabolic, antitumor, antimicrobial, anti-inflammatory, and immunomodulatory properties [4].

*Arnica montana* L. (mountain arnica) is a very old medicinal plant growing on mountain slopes in Europe, northern Asia, Siberia, and America. The plant contains over 150 biologically active substances; the main among them are sesquiterpene lactones helenalin and dihydrohelenalin and their ester derivatives, phenolic acids, flavonoids, carotenoids, essential oils, terpenoids, polysaccharides, and pyrrolizidine alkaloids [5]. *A. montana* possesses significant anti-inflammatory, antibacterial, antifungal, antioxidant, and immunomodulatory activity and is used traditionally to treat various ailments: contusions, wounds, rheumatism, and inflammation [5,6].

*Stevia rebaudiana* Bertoni, a perennial shrub also known as “Honey Leaf”, “Sweet Leaf”, or “Sweet Herb” is a great alternative to synthetic sweeteners being approximately 200 to 300 times sweeter than sugar [7]. Stevioside and rebaudioside A are the two main diterpenoid steviol glycosides, to which the sweet taste of the plant is due. The other compounds found in stevia leaves are flavonoids, alkaloids, hydroxycinnamic acids (caffeic, chlorogenic, etc.), oligosaccharides, free sugars, amino acids, and lipids [8]. The plant possesses several biological activities including anti-hypertensive, anti-obesity, antidiabetic, antioxidant, anticancer, anti-inflammatory, and antimicrobial effects [9].

*Echinacea purpurea* (L.) Moench (echinacea or purple coneflower) is a perennial medicinal plant that contains different classes of secondary metabolites such as alkamides, caffeic acid derivatives, polysaccharides, and glycoproteins [10]. The herb is one of the most well-known and widely used therapeutic plants worldwide. The plant has important immunostimulatory and anti-inflammatory properties and is used in a diverse range of herbal products [11].

*Cichorium intybus* L. (chicory) contains sesquiterpene lactones (particularly lactucin, lactucopicrin, 8-deoxy lactucin, and guaianolid glycosides, including chicoroisides B and C, sonchuside C), inulin, sugars, proteins, hydroxycoumarins, flavonoids, alkaloids, steroids, terpenoids, oils, volatile compounds, coumarins, vitamins, and polyynes. Chicory exhibits different biological activities including gastroprotective, cardiovascular, antioxidant, hypolipidemic, anticancer, antidiabetic, anti-inflammatory, analgesic, sedative, immunological, antimicrobial, anthelmintic, anti-protozoal, wound healing, and many other pharmacological effects [12,13].

*Silybum marianum* (L.) Gaertn. or milk thistle, has long been used for its liver-protective properties. The plant is native to the Mediterranean area and is now growing and cultivated worldwide. *S. marianum* fruits contain a mixture of flavonolignans collectively known as silymarin - silybin, silydianin, and silycristin [14]. Silymarin, with its strong antioxidant properties, has been proven to promote liver cell regeneration, reduce blood cholesterol, and help prevent cancer [15].

*Tanacetum parthenium* (L.) Sch.Bip. (feverfew) contains mainly sesquiterpene lactones parthenolide with strong analgesic, anti-inflammatory, and antipyretic activities [16]. The species is a short, bushy, aromatic perennial plant native to Kazakhstan, Central Asia, and the Mediterranean region and is now distributed in Europe, Asia, and America.

Attempts to cultivate some of these medicinal plants have been made since ancient times. The secondary metabolite production in plants is often low (less than 1% dry weight) and is highly dependent on the physiological and developmental stage of the plant [17,18]. The increased demand for these phytochemicals requires a production platform that ensures a continuous supply, homogeneous production, and low environmental impact [19]. Plant tissue culture is an alternative approach for the large-scale bio-production of valuable secondary products, and it can meet all the requirements mentioned above. In vitro plant cell and tissue culture techniques including micropropagation, cell suspension, and organ cultures (shoots and roots) represent a good platform for biomass accumulation and the synthesis of desired substances. In addition, hairy root cultures that were obtained by the infection of plant explants with *Agrobacterium rhizogenes* are also effective biotechnological tools for secondary metabolite accumulation.

Elicitation has been extensively employed in in vitro plant cell and tissue culture to induce the synthesis of secondary metabolites or to initiate their de novo synthesis [20]. The successful production of secondary metabolites is influenced by several parameters, including the elicitor concentration, selectivity, duration of exposure, age of culture, cell line, growth regulation, nutrient composition, and quality of cell wall materials [21].

## 2. Elicitors

**Figure 1:**
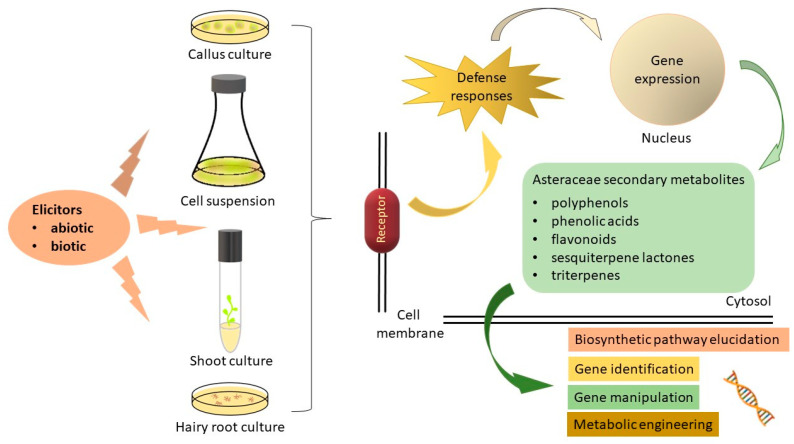
A graphical overview of the biotechnological strategies for secondary metabolite synthesis by elicitation.

Elicitors are signals or molecules that trigger the formation of biologically active secondary metabolites. The elicitation process arises in response to stress stimuli that activate the protective mechanisms of plants through secondary metabolite biosynthesis [22]. The exogenous elicitors usually act together with plant membrane receptors and initiate signal transduction pathways that lead to the transcriptional stimulation of different genes and induce the synthesis of various molecules involved in the protection or resistance of plants [21,23,24]. Secondary metabolite production increases in the plant system due to the stress induced by the employment of the elicitors [25]. The use of elicitors for the production of multiple biologically active secondary metabolites has been reported in many plant species [26,27]. Depending on their nature and origin, the elicitors are divided into biotic and abiotic (Figure 1).

Abiotic elicitors are substances of non-biological origin and are divided into physical, chemical, and hormonal factors [28]. Biotic elicitors are chemicals with a biological origin, such as microorganisms and polysaccharides found in plant cell walls (such as chitin, pectin (PEC), and cellulose). Light, osmotic stress, salinity, drought, and temperature stress are examples of physical elicitors. Chemical elicitors include heavy metals, metal ions, nanoparticles, and inorganic salts. Elicitation research has made considerable use of a variety of plant hormones. The most used plant growth regulators (PGR) are jasmonate, salicylic acid, and gibberellic acid (GA). Jasmonic acid (JA) and its associated growth-regulating signal molecules, jasmonate, and salicylic acid (SA) are one of the most commonly used elicitors which are produced in plants in response to abiotic or biotic stress and lead to phenol, alkaloid, carotenoid, and chlorophyll accumulation [29,30]. In terms of its high efficiency, low expenses, and easy technique, elicitation is a potential strategy for enhancing the production of plant secondary metabolites [31]. It has been found that the application of different abiotic and biotic elicitors promotes the in vitro shoot multiplication of various plant species [32,33,34]. The treatment of the in vitro cultures of medicinal plants with abiotic and biotic elicitors showed different responses to the same chemical elicitors. Additionally, the best elicitor type and concentration varied greatly depending on the target bioactive compounds and the plant species. Thus, it is essential to optimize the elicitor type and concentration for each purpose [24].

### 2.1. Abiotic Elicitors

#### 2.1.1. Physical Elicitors

Light is an important factor for normal growth, organogenesis, and the production of primary and secondary metabolites. Different light sources, including UV, fluorescent, and LEDs, have been reported as elicitors of secondary metabolite production [35]. The impact of light irradiation on the production of artemisinin in *A. annua*’s hairy roots was studied by Liu et al. [36]. The authors found that growth and artemisinin accumulation increased with improved conditions of light irradiation, and hairy roots grew at the slowest rate in the dark (Table 1). The control white light improved the callogenic frequency and biomass accumulation of *S. rebaudiana*, while blue light enhanced phenolic and flavonoid contents [37]. An enhanced accumulation of phenolics has been reported in light-grown suspension cultures of *Artemisia absinthium* [38], while the green spectrum was more beneficial for total flavonoids, total phenolics, and antioxidant activity in callus cultures of the same species [39]. Under red light, silychristin, isosilychristin, silydianin, silybin A, and silybin B levels of *S. marianum* callus culture have been the highest, whereas isosilybin A and isosilybin B content has been found to be the highest under green light [40]. The authors reported that the accumulation of taxifolin was optimal under continuous white light. Numerous studies have shown that plant responses to light depend on the plant species, light source and quality, culture conditions, etc. The caffeic acid and antioxidant activity of callus cells, also growth parameters, total phenol content, and the antioxidant activity of the cell suspensions of *E. purpurea* have all been raised by all UV-B treatments in a dose-dependent manner [41]. Continuous light for 14 days significantly increased the levels of certain caffeic acid derivatives in cell suspensions of *Echinacea angustifolia* and reduced the level of hydroxytyrosol derivatives with rhamnose residues [42]. Similar results have been reported in the light-grown hairy roots of *E. purpurea*. The authors observed increased levels of anthocyanins and caffeic acid derivative’s biosynthesis, which correlated with the light-stimulated activity of phenylalanine ammonium lyase [43].

Blue LED light improved the seed germination of *S. rebaudiana* and had an impact on the development of the greatest number of leaves and roots in 4-week-old in vitro plantlets. The highest fresh weight of stevia plantlets was attained at 20 °C under combined red and white LED light and at 25 °C under white, fluorescent light. The high accumulation of phenolics and soluble sugars was achieved in plantlets growing in the darkness and irradiated blue LED light. The activity of antioxidant enzymes has been impacted by all LEDs [44]. The other authors have also observed that the spectrum of different light sources affects in vitro morphogenesis, the proliferation of shoots, growth, and the rooting of *S. rebaudiana* [45]. Red LED light treatments increased the multiplication rate of stevia shoots compared with the control [45,46,47]. The violet light showed the maximum accumulation of fresh biomass in the adventitious root culture of *S. rabaudiana* and the highest 2,2-diphenyl-1-picrylhydrazyl (DPPH) free radical scavenging activity inhibition, while the blue light enhanced the phenolic content and total flavonoid production [48]. The cultivation of *S. rebaudiana* plantlets using an LED source with an intensity of 75 and 230 µmol/(m^2^s) had a beneficial effect on the development of plantlets with a combination of morphological and mesostructural parameters important for reproduction under in vitro conditions and subsequent ex vitro adaptation [49]. It has been demonstrated that the far-red LED induction system in TIS RITA^®^ has a favorable impact on the growth of stevia shoots and increases the production of metabolites (stevioside and rebaudioside-A) by up to 37.15% and 22.99%, respectively [50]. Lighting with radiation equalized by the flux density of photosynthetic photons and ratios of radiation levels in the region of FR—far red > R—red has been found to promote callus fresh weight and inulin content in callus cells of *Chicorium intybus* [51]. Yoneda et al. [52] reported that red/far-red 1.22 and blue lights increased the transcription of *UGT85C2* (one of the UDP-glycosyltransferases involved in catalyzing the sugar transfer reaction) and led to a higher accumulation of steviol glycosides in *S. rebaudiana* grown hydroponically.

##### Osmotic Stress

One of the major environmental stimuli that can change the physiological and biochemical characteristics of plants and raise the concentration of secondary metabolites in plant tissues is osmotic stress (also known as water stress). In plant cell cultures, osmotic agents such as sucrose, glucose, and polyols (sugar alcohols) can be used to create osmotic stress and increase the synthesis of secondary metabolites [53]. The higher concentrations of proline and polyethylene glycol (PEG) in the culture media are used to induce osmotic stress in plant tissue culture. The application of higher doses caused a variety of metabolic alterations that led to water deficits and drought stress. Proline acts as an osmolyte and has different functions as a cytoplasmic enzyme protector, a source of carbon and nitrogen for post-stress growth, and even a stabilizer of the machinery involved in protein synthesis, cytosolic acidity management, and free radical scavenging [54]. PEG is a non-ionic water polymer that has been employed in vitro to cause water stress in plants; however, it is not expected to quickly permeate plant tissue. PEG is a substance used to keep a nutrient medium’s osmotic potential equal to that of the cultured cells (i.e., the medium and the cells are isotonic). This osmotic balance prevents in vitro cell damage. Proline- and PEG-treated *S. rebaudiana* callus and suspension culture showed increased steviol glycoside synthesis [55]. Among the osmolytes, sucrose is a carbon source that has been shown to effectively enhance the formation of secondary metabolites in a wide variety of elite plants cultivated in vitro [56]. The enhanced growth and biomass accumulation of *A. montana* hairy roots is achieved on Murashige and Skoog (MS) nutrient medium containing 3% or 5% sucrose. Gas Chromatography-Mass Spectrometry analysis showed the presence of flavones, phenolic acids, organic acids, fatty acids, amino acids, sugars, sugar alcohols, hydrocarbons, etc. [57].

Sorbitol and mannitol raise osmotic potential in the medium solely and were not applied as nutritional sources in plant cell cultures. These compounds establish conditions of osmotic stress in plants that lead to a reduction in plant water uptake [58]. The sorbitol treatment enhanced the malondialdehyde and hydrogen peroxide content. It caused oxidative stress in cell suspension cultures of *A. annua*. In contrast, the application of coronatine and sorbitol together increased the expression of artemisinin biosynthetic genes and artemisinin production at all tested concentrations [53]. The successful protocol for the slow-growth storage of in vitro *A. montana* shoot cultures is reported after testing eight different treatments with mannitol or sorbitol, which maintain plant quality for up to 6 months [59]. The preservation of the *Artemisia herba-alba* Asso microshoots on a medium supplemented with 0.1, 0.2, or 0.3 M sucrose, mannitol, or sorbitol has been found to inhibit the growth rate and maintain shoots for up to 12 weeks [58].

The salt content of the culture medium has an osmoregulatory effect that impacts cell development and morphogenesis in addition to its nutritional effects. The effect of different strengths of MS medium (full, half-, third-, and quarter-content salts and vitamins) on the micropropagation, shoot growth, in vitro rooting, and antioxidant properties of *A. montana* has been tested by Zayova et al. [60]. It was found that the highest phenolic content and antioxidant activity were obtained in shoots grown on the quarter strength of MS medium, whether derived from micropropagation or the rooting stage. Reducing the macro- and micro-salt content of the nutrient medium resulted in a low propagation rate. Decreased salt concentration causes osmotic stress and induces the production of antioxidant metabolites.

##### Drought Stress

Drought stress is an abiotic stress that influences plant growth and development. Drought stress generally causes plants to absorb less water, depriving them of vital nutrients and thus retarding their growth and development [61]. Drought also affects the yield, membrane integrity, pigment content, osmotic adjustment, and photosynthetic activity of plants [62]. Reactive oxygen species (ROS), which are produced during drought stress, cause damage to membranes, DNA, lipids, and amino acids and disrupt the activities of several enzymes [63]. It has been reported to increase the accumulation of flavonoids and phenolic acid in plants under drought stress. The morphological, physiological, and antioxidant responses of *S. rebaudiana* under in vitro agar-induced drought stress have been studied [64]. The highest accumulation of proline content (50%) and malondialdehyde (MDA) (42%) are recorded in the highest concentration of agar at 14 g/L, but antioxidant enzyme activities such as ascorbate peroxidase, catalase, and peroxidase are enhanced by the addition of 8 g/L agar to the cultural medium. The plant growth retarding and root induction inhibition of *S. marianum* in vitro seedlings under mannitol-induced drought stress were reported [65]. However, total phenolic content, total flavonoid content, and total protein content were found to be positively regulated with an increasing degree of drought stress. The chalcone synthase genes’ (*CHS1*, *CHS2*, and *CHS3*) expression in milk thistle, which are associated with the silybin biosynthetic pathway, as well as transcription levels of genes encoding antioxidant enzymes *APX*, *SOD*, *CAT*, *1-Cys-Prx*, and *PrxQ*, were increased under drought stress [66]. De Caroli et al. [67] reported the important role of the *CiXTH29* and *CiLEA4* genes as potential markers for drought stress tolerance in *C. intybus* varieties. Earlier, it was found that the fresh and dry root weight, leaf number, total leaf area, stomatal conductance, and inulin yield were all decreased under water stress in chicory [68]. Water deficits induced oxidative stress and the expression of proline metabolic genes but reduced the growth, biomass accumulation, and chlorophyll content of *A. annua* grown under greenhouse conditions [69]. The authors found that the artemisinin content of *A. annua* might be regulated by controlling irrigation regimes.

##### Salinity

Salinity affects numerous physiological and metabolic processes, inhibits plant growth and development, and affects differentiation levels as well as primary metabolisms including photosynthesis, protein synthesis, energy production, and lipid metabolism in plants [70,71,72]. Plants subjected to physiological water deficiency due to salinity are at risk of oxidative stress associated with the overproduction of ROS (reactive oxygen species) that damage nucleic acids, proteins, and lipids [73]. The accumulation of secondary metabolites, such as phenols, terpenes, and alkaloids, is known to be induced or stimulated by exposure to salinity [74,75,76]. Treatment with NaCl negatively affected root numbers and weights (dry and fresh) of in vitro cultured *S. rebaudiana* Bertoni but increased hydroxycinnamic acid accumulation, antioxidant capacity, and total soluble sugar content. In addition, there has been evidence that NaCl treatment induced changes in the expression of steviol glycoside-related biosynthetic genes [77]. According to Javed and Gürel [78], the in vitro growth of *S. rebaudiana* calli and shoots revealed significant salt tolerance by calli and shoots at up to 100 mM NaCl concentration supplemented with an MS nutrient medium. The steviol glycosides, such as rebaudioside A and stevioside, as well as the total phenolic content, total flavonoid content, total antioxidant capacity, total reducing power, and DPPH free radical scavenging activity, all showed a significant increase under treatment with 100 mM NaCl. By studying two varieties of *S. rebaudiana* Bertoni in in vitro conditions, it has been found that polyphenol oxidase and malate dehydrogenase enzyme activities and gene expression can be used as biochemical and molecular markers to detect the resistance or susceptibility nature of stevia cultivars against salinity [79]. The application of the same concentration of 100 mM NaCl or KCl to one-month-old *Artemisia arborescens* L. in vitro microshoots led to an improvement in the accumulation of total polyphenols, flavonoids, condensed tannins, and volatile compounds [80]. Salinity also has a positive effect on the accumulation of secondary metabolites in plants of the Moringaceae family [81]. The presence of NaCl in the MS nutrient medium leads to a significant increase in the phenolic acids and flavonoid content of Moringa Callus.

**Table 1:** Effect of physical elicitors on biomass accumulation and secondary metabolite content in in vitro culture of Asteraceae plants.

Plant Species	Physical Elicitors	Type of In Vitro Culture	Effects	References
	**UV radiation**			
*Artemisia annua* L. (sweet wormwood)	Light irradiation (3000 Lux for 16 h cool-white fluorescent lamps and then darkness for 8 h)	HR	Hairy roots’ growth and artemisinin accumulation increased.	[36]
*Artemisia absinthium* L. (common wormwood)	Different spectral lights	CC	Red spectrum enhanced peroxidase activity, protease activity, total protein content, and chlorophyll a/b ratio; green spectrum stimulated TPC, TFC, and AA; yellow light enhanced MDA content, while white and green light improved total chlorophyll content and carotenoid content.	[39]
*Artemisia absinthium* L. (common wormwood)	Light	SuspC	Maximum increase in dry biomass; high AA; enhanced levels of TPC and total secondary metabolites in light-grown suspension cultures during log phase of growth.	[38]
*Stevia rebaudiana* Bert. (stevia)	Different spectral lights	CC	Control white light improved callogenic frequency and biomass accumulation; blue light enhanced TPC and TFC; green and red light improved AA.	[37]
*Stevia rabaudiana* Bert. (stevia)	Light-emitting diodes’ (LEDs’) spectra	ShC	Blue LED light increased seed germination. Blue LED light stimulated the growth of roots and leaves of in vitro plantlets; and the number and opening of stomata. Red LED light increased stems and roots, but it had the least positive impact on the production of carotenoids and chlorophylls. Red and blue LED light have opposite effect on the activity of antioxidant enzymes (catalase (CAT), peroxidase (POD), and superoxide dismutase (SOD)).	[44]
*Stevia rabaudiana* Bert. (stevia)	Light-emitting diodes’ (LEDs’) spectra	ShC	Red LEDs enhanced proliferation rate; Blue/Red LED promoted shoot elongation.	[45]
*Stevia rabaudiana* Bert. (stevia)	Red LED	ShC	*S. rebaudiana* microshoots grown on MS media with 1.0 mg L^−1^ 6-benzylaminopurine (BAP) and 0.5 mg L^−1^ Indole acetic acid (IAA) under red monochromatic light treatments showed a 30% increase in multiplication coefficient compared to control (white light, media without PGRs).	[46]
*Stevia rabaudiana* Bert. (stevia)	Light-emitting diodes’ (LEDs’) spectra	ShC	Blue LED light promoted shoot elongation, leaves number/shoot, leaf fresh weight, leaf dry weight, and photosynthetic pigment production; red LED treatment resulted in high shoot production; fluorescent light induced 100% root induction.	[47]
*Stevia rabaudiana* Bert. (stevia)	Spectral lights	AdvRC	Violet light showed maximum FW and highest DPPH inhibition, while blue light enhanced phenolic content and total flavonoid production.	[48]
*Stevia rabaudiana* Bert. (stevia)	LED treatment	ShC	LED source with an intensity of 75 and 230 µmol/(m^2^ s) enhanced FW accumulation of aerial parts and roots and improved ex vitro adaptation of plants.	[49]
*Stevia rabaudiana* Bert. (stevia)	Far-red LED induction system	ShC	Favorable impact on the growth of shoots and production of metabolites (stevioside and rebaudioside-A) by up to 37.15% and 22.99%, enhancing gene expression related to steviol glycosides.	[50]
*Cichorium intybus* L. (common chicory)	Artificial Light	CC	Radiation levels in the region of FR—far red > R—red promote callus fresh weight and inulin content in callus cells.	[51]
*Silybum marianum* L. (milk thistle)	Monochromatic lights	CC	High levels of silychristin, isosilychristin, silydianin, silybin A, and silybin B under red light; maximum content of isosilybin A and isosilybin B under green light; high amount of taxifolin under continuous white light.	[40]
*Echinacea purpurea* L. (echinacea)	UV-B treatments	CC; SuspC	Enhanced caffeic acid and growth parameters, TPC, and AA of cell suspensions.	[41]
*Echinacea angustifolia* DC. (blacksamson echinacea)	Continuous light	SuspC	Continuous light for 14 days significantly increased levels of certain caffeic acid derivatives and reduced level of hydroxytyrosol derivatives with rhamnose residues.	[42]
*Echinacea purpurea* L. (echinacea)	Light	HR	Increased levels of anthocyanins and caffeic acid derivative biosynthesis, stimulated the activity of phenylalanine ammonium lyase.	[43]
	**Osmotic stress**			
*Stevia* *rebaudiana* Bert. (stevia)	Proline and PEG	CC;SC	Increased steviol glycoside synthesis.	[55]
*Arnica montana* L. (mountain arnica)	Sucrose Maltose Glucose	HR	Enhanced growth and biomass accumulation of *Arnica montana* hairy roots on MS nutrient medium containing 3% or 5% sucrose; sugars and sugar alcohol content were influenced by the concentration of respective carbon sources in the nutrient medium.	[57]
*Arnica montana* L. (mountain arnica)	Sorbitol and mannitol	ShC	Reduced plant growth; maintain plant quality for up to 6 months.	[59]
*Artemisia annua* L. (sweet wormwood)	Sorbitol and coronatine	SuspC	Sorbitol treatment enhanced malondialdehyde and hydrogen peroxide content; application of coronatine and sorbitol together increased the expression of artemisinin biosynthetic genes and artemisinin production at all tested concentrations.	[53]
*Arnica montana* L. (mountain arnica)	Medium salt content	ShC	The highest phenolic content and antioxidant activity are obtained in shoots grown on ¼ strength of MS medium.	[60]
	**Drought stress**			
*Stevia rebaudiana* (stevia)	Agar-induced drought stress	ShC	The highest accumulation of proline and malondialdehyde are detected in the highest concentration of agar, but enhanced antioxidant enzyme activities are obtained on 8 g/L agar containing cultural medium.	[64]
*Silybum marianum* (milk thistle)	Mannitol- induced drought stress	ShC	Inhibited root induction; retarded plant growth; enhanced accumulation of TPC, TFC, and total protein content along with several antioxidative enzymes.	[65]
	**Salinity**			
*Stevia rebaudiana* Bert. (stevia)	NaCl	ShC	Increased AA, hydroxycinnamic acid, and total soluble sugar content; induced changes in expression of steviol glycoside-related biosynthetic genes.	[77]
*Stevia rebaudiana* Bert. (stevia)	NaCl	CC, ShC	Steviol glycosides rebaudioside A and stevioside, as well as TPC, TFC, and AA, showed significant improvement.	[78]
*Stevia rebaudiana* Bert. (stevia)	NaCl concentrations: 500, 1000, 2000, and 3000 mg L^−1^	CC, ShC	Salinity has a negative effect on shoot number, shoot length, root number, root length, leaf number, and total chlorophyll content; biochemical markers peroxidase (PDO), polyphenol oxidase (PPO), and malate dehydrogenase (MDH) were associated with salt tolerance.	[79]
*Artemisia arborescens* L. (wormwood)	NaCl or KCl	ShC	Improvement in TPC, TFC, condensed tannins, and volatile compounds accumulation.	[80]

Legend: AdvRC—adventitious root culture; CC—callus culture; HR—hairy root culture; ShC—shoot culture; SuspC—suspension culture; TPC—total phenolic content; TFC—total flavonoid content; and AA—antioxidant activity.

#### 2.1.2. Plant Growth Regulators

##### Jasmonic Acid and Methyl Jasmonate

Jasmonic acid occurs in higher plants and is responsible for plant growth and development in general and during biotic and abiotic stress [82]. Jasmonate plays a crucial role in the elicitation process that results in de novo transcription and translation, which in turn enhances the production of secondary metabolites in in vitro plants [83]. Methyl jasmonate (MeJA), a volatile jasmonic acid methyl ester, has been found to be a key signalling molecule in both biotic and abiotic stressors [84]. MeJA is considered an important phytohormone that can enable intra- and inter-communications in plants, controlling defense responses, especially antioxidant systems, because of its volatile nature and ability to permeate biological membranes. Exogenous MeJA application to plant in vitro cultures has been recognized as a unique strategy for improving secondary metabolite production, upregulating antioxidant enzyme activity, and expressing defense-related genes [85,86].

The exogenous application of methyl jasmonate induces the expression of plant genes for various biosynthetic pathways [83,87]. Baldi and Dixit [88] have reported a significant increase in artemisinin (sesquiterpene lactone) in the suspension cell cultures of *A. annua* after treatment with MeJA (Table 2). A similar stimulating effect has been observed in plants and the hairy roots of *A. annua* [89,90]. In addition to increasing the content of artemisinin, the expression of the gene *CYP71AV1* coding enzyme monooxygenase, which catalyzes the oxidation of amorpha-4,11-diene to artemisinic acid, was also increased. Therefore, the *CYP71AV1* gene regulated at the transcript level by MeJA may be a suitable candidate for metabolic engineering strategies to increase the content of artemisinin [91]. In some cases, better results for the synthesis of biologically active substances are obtained when MeJA is combined with some supplements such as precursors or in combination with an abiotic elicitor (Table 2). An integrated yield enhancement strategy for artemisinin production by suspension cultures of *A. annua* has been developed [88]. The authors used mevalonic acid lactone as a precursor and MeJA as an elicitor and achieved a 15.2 g/L biomass and 110.2 mg/L artemisinin yield, which is 5.93 times higher in comparison to control cultures. A similar strategy has been applied to the cultivation of the hairy roots of *A. annua*; however, the authors used MeJA (40 μg/L) and the precursors casein acid hydrolysate (50 μg/L) and sodium acetate (500 μg/L) to achieve a maximum artemisinin content of 3.45 mg/g on 15 days [92]. Studies confirmed the positive effect of a combination of chemically modified β-cyclodextrin (50 mM) and MeJA (100 μM) treatments on artemisinin accumulation in suspension cultures of *A. annua*. About 300 times more artemisinin (27 μmol g^−1^ dry weight) was found in comparison with suspensions that have not been treated [93]. Hairy root treatment with MeJA (200 μM) increased artemisinin yield to 1.52 μg mg^−1^ of dry weight [94].

The enhancement of 2.44 times in artemisinin concentration in the hairy roots of *A. annua* by the combined addition of MeJA (100 μM) and cell homogenate of *Piriformospora indica* (3% *v*/*v*) in the culture medium has been reported [95]. These elicitors upregulated the genes of MVA, MEP, and artemisinin biosynthetic pathways, viz. *hmgr*, *ads*, *cyp71av1*, *aldh1*, *dxs*, *dxr*, and *dbr2*. Silymarin accumulation was enhanced in cell suspension by adding in the culture medium of MeJA (100 μM) [96] or MeJA (100 μM), YE (50 μg/mL), and Phe (0.1 mM) [97] and responded to MeJA treatment in a dose-dependent manner [98]. Increasing the MeJA concentrations significantly raised the total silymarin content in the hairy roots of *S. marianum*. Earlier, it was reported that silymarin in cell suspension culture increased after treatment with MeJA and methyl B cyclodextrin [99]. It was found that the MeJA concentration of 7.5 mg/L was optimum for the elicitation of caffeic acid derivatives, in particular chicoric acid, produced by *E. purpurea* cell culture [100]. In addition, a suitable gene marker *C*_3_*H*, p-coumarate-3-hydroxylase, useful for early predictions of the cell’s aptitude to synthesize these molecules, has been identified [100]. MeJA treatments raised the caffeic acid derivatives (CADs) in both shoots and roots in *E. purpurea* applied with 100 μM MeJA and collected 45 days after application, but other growth parameters of the shoot and roots declined in line with the rising level of MeJA treatments [101]. Cui et al. [102] reported that MeJA treatment (100 μM) in a 500 L pilot-scale bioreactor culture system in adventitious roots of *E. angustifolia* generated 12.3 mg/g DW echinacoside. The echinacoside content was over twice that of the control group [102]. MeJA treatments had a detrimental impact on biomass and growth indexes in all callus, shoot, and root cultures of *E. purpurea*. However, the treatments led to a rise in total phenolics and CAD accumulation, particularly at concentrations of 100 and 150 µM MeJA [103]. The 25 μM MeJA treatment increased metabolite production in adventitious roots of *E. purpurea* and *E. pallida* with the maximum production of phenolics (728.2 mg/L), flavonoids (622.2 mg/L), and caffeic acid derivatives (255.3 mg/L cichoric acid and 143.9 mg/L echinacoside); however, the highest polysaccharide production (approximately 440 mg/L) has been determined at 50 to 200 μM MeJA [104]. The biosynthesis of the sesquiterpene lactones in the hairy roots of *C. intybus* has been upregulated by MeJA (highest after 72 h) [105]. The application of MeJA to chicory hairy roots increases 3,5-dicaffeoylquinic acid accumulation [106]. The influence of MeJA and JA and a phytohormone gibberellic acid applied at different concentrations on growth kinetics, secondary metabolites production, and antioxidant activity in cell suspension cultures of *A. absinthium* was studied. The dry biomass accumulation has been inhibited by the application of elicitors, but an enhanced accumulation of the total phenolic content, total flavonoid content, and highest radical scavenging activity in suspension cultures treated with 1.0 mg/L of MeJA, JA, and GA have each been noticed [107]. An improved accumulation of essential oils through elicitation on adventitious roots of *A. amygdalina* with methyl jasmonate (0.5 mg/L) was obtained. The treatment resulted in higher production of total phenolic content, total flavonoid content, and phenylalanine ammonia lyase activity [108]. The combined elicitors (2.5 mg/L YE and 100 µM MeJA) enhanced parthenolide production and the expression of the parthenolide synthase (TpPTS) gene in *Tanacetum parthenium* hairy roots [109]. The same elicitors yeast extract (YE) and MeJA applied for 48 h in a cell suspension of *T. parthenium* grown in MS medium supplemented with 0.5 mg/L 2,4-D and 0.1 mg/L TDZ have been found to induce parthenolide synthesis [110]. An increase in the expression level of the MEP pathway genes (*DXS*, *CMS*, *CMK*, *HDS*, and *HDR*) under the effect of MeJA in *S. rebaudiana* in vitro plants grown in the continuous-flow hydroponic system has been reported [111]. The authors noticed that MeJA has a considerable effect on activating 12 (80%) of the genes that encode the enzymes involved in the steviol glycosides’ biosynthesis pathway, which makes it a promising elicitor for optimizing the steviol glycosides’ accumulation process.

##### Salicylic Acid

Salicylic acid is a signalling molecule with roles in plant growth and development that regulate the expression of various stress-related genes, leading to the de novo synthesis of some important proteins involved in conferring tolerance to the plant [112]. Moharramnejad et al. [113] tested the effect of MeJA and SA on in vitro growth, stevioside production, and the oxidative defense system in micropropagated *Stevia rebaudiana* plants. The authors observed optimum shoot growth, root regeneration, and fresh and dry biomass accumulation on woody plant medium (WPM) supplemented with 50 μM SA. The WPM containing MeJA and SA decreased CAT activity and increased total phenolics, total antioxidant activity (DPPH), and SOD and POX activities, particularly those of SOD2 and POX1 isoforms [113]. The supplementation of MS medium with 0.25 mg/L SA resulted in high rebaudioside A content (3.40 mg/g dry weight callus), while an SA concentration of 0.75 mg/L promoted the callus growth rate (0.1 cm/day), callus diameter (0.79 cm), and relative callus fresh weight (0.085 g) [114]. The foliar application of SA led to improvements in chlorophyll and carotenoid contents, a significant enhancement in the net photosynthetic rate (31.7%), the activity of nitrate reductase (17.2%) and carbonic anhydrase (10.9%), and the content and yield of artemisinin [115]. Recently, it has been reported that SA induces artemisinin biosynthesis in at least two ways: by increasing the conversion of dihydroartemisinic acid into artemisinin caused by the burst of ROS and by upregulating the expression of genes involved in artemisinin biosynthesis [90]. SA pretreatment decreased mitigated adverse effects of PEG-simulated drought stress on *A. aucheri* under in vitro conditions by improving the activity of antioxidant enzymes [116]. The effects of SA as an elicitor and tyrosine as a precursor on propagation and some secondary compound production in *E. purpurea* in vitro are tested. The results indicated that the 2 mg/L SA treatment was superior to the other treatments, resulting in the highest average number of branches formed (33.6 branches/plant part), leaves (33.6 leaves/vegetable part), fresh 1.067 g and dry 0.058 g weights, and a 291.3427 μg/g DW concentration of echinacoside [117].

Comparing the influence of elicitors, in particular *SA*, on second metabolite accumulation in plants from the other plant family, it is established that SA affects differently [81]. For example, adding salicylic acid at a concentration of 200 μM to the MS nutrient media led to an increase in total phenolic compounds in the callus of *Moringa oleifeira* from the Moringaceae family, after 15 and 30 days of treatment.

**Table 2:** Effect of some plant growth regulators on biomass accumulation and secondary metabolite content of Asteraceae plants.

Plant Species	Elicitors	Type of In Vitro Culture	Effects	References
	Plant growth regulators			
*Artemisia annua* L. (sweet wormwood)	MeJA and mevalonic acid lactone as a precursor	SuspC	5.93 times higher artemisinin productivity compared to control cultures	[88]
*Artemisia annua* L. (sweet wormwood)	MeJA (40 μg/L) and the precursors casein acid hydrolysate (50 μg/L) and sodium acetate (500 μg/L)	HR	A maximum artemisinin content of 3.45 mg/g on 15 days after treatment	[92]
*Artemisia annua* L. (sweet wormwood)	β-cyclodextrin (50 mM) and MeJA (100 μM)	SuspC	About 300 times more artemisinin than the control	[93]
*Artemisia annua* L. (sweet wormwood)	MeJA (100 μM) and cell homogenate of *Piriformospora indica* (3% *v*/*v*)	HR	The enhancement of 2.44 times in artemisinin concentration in the hairy roots; positive correlation with regulatory genes of the MVA, MEP, and artemisinin biosynthetic pathways, viz. *hmgr*, *ads*, *cyp71av1*, *aldh1*, *dxs*, *dxr*, and *dbr2*	[95]
*Artemisia annua* L. (sweet wormwood)	Twenty-two micromolar MeJA	SuspC	Three-fold increase in artemisinin production in around 30 min; the MeJA-induced upregulation of *CYP71AV1*	[91]
*Silybum marianum* L. (milk thistle)	MeJA and methyl B cyclodextrin	SuspC	Silymarin accumulation increased	[99]
*Silybum marianum* L. (milk thistle)	MeJA	CC, HR	Increase the total silymarin	[98]
*Silybum marianum* L. (milk thistle)	MeJA	CC	MeJA promoted the accumulation of silymarin and enhanced chalcone synthase (CHS) activity	[96]
*Silybum marianum L. (milk thistle)*	MeJA (100 μM), YE (50 μg.mL^−1^), and Phe (0.1 mM)	CC	The enhancement of the silymarin production - 8.6 times higher than the control	[97]
*Echinacea purpurea* L. (echinacea)	MeJA 7.5 mg/L	SuspC	Chicoric acid accumulation; *C_3_H* expression appeared correlated with the enhanced biosynthesis of chicoric acid in *E. purpurea* cell suspension and significantly boosted by MeJA elicitation	[100]
*Echinacea purpurea* L. (echinacea)	MeJA 100 μM	ShC	Raised the caffeic acid derivatives in both shoots and roots; growth parameters of the shoot and roots declined in line with the rising level of MeJA treatments	[101]
*Echinacea purpurea* L. (echinacea)	MeJA	CC; ShC; RC	Rise in TPC and CAD accumulation, particularly at concentrations of 100 and 150 µM MeJA; inhibited biomass and growth indexes in all callus, shoot, and root cultures	[103]
*Echinacea purpurea* L. (echinacea) and *E. pallida* Nutt. (pale purple coneflower)	25 μM MeJA	AdvRC	The maximum production of phenolics, flavonoids, and caffeic acid derivatives (cichoric acid and echinacoside); maximum levels of activities of antioxidant enzymes (superoxide dismutase, peroxidase, ascorbate peroxidase, and catalase) with 25 μM MeJA	[104]
*Cichorium intybus* L. (common chicory)	MeJA 100 μM	HR	High sesquiterpene lactone accumulation	[105]
*Cichorium intybus* L. (common chicory)	MeJA	HR	High 3,5-dicaffeoylquinic acid; the biomass of hairy roots and rates of CQAs are higher than in plants and other hairy root cultures	[106]
*Artemisia absinthium* L. (common wormwood)	MeJA, JA and gibberellic acid (GA)	SuspC	Biomass accumulation was inhibited by the application of elicitors but enhanced the accumulation of TPC, TFC, and high AA	[107]
*Artemisia amygdalina* Decne.	Methyl jasmonate	AdvRC	The higher production of TPC, TFC, and phenylalanine ammonia lyase activity	[108]
*Tanacetum parthenium* L. (feverfew)	2.5 mg/L YE and 100 µM MeJA	HR	Enhanced parthenolide production and the expression of the parthenolide synthase (*TpPTS*) gene	[109]
*Tanacetum parthenium* L. (feverfew)	2.5 mg/L YE and 0.5 mg/L MeJA	SuspC	The highest parthenolide accumulation is achieved in the cell suspension containing 0.5 mg/L 2,4-D and 0.1 mg/L TDZ treated with YE + MeJA elicitor for 48 h	[110]
*Stevia rebaudiana* Bert. (stevia)	Salicylic acid (SA) and MeJA	ShC	The maximum shoot growth, root regeneration, and FW and DW accumulation on WPM containing 50 μM SA; WPM containing MeJA and SA increased SOD2 and POX1 activity, TPC, and AA and decreased CAT activity; the highest levels of enzymatic and non-enzymatic antioxidants were observed in the WPM containing 100 μM SA; 50 μM MeJA and 100 μM SA enhanced stevioside production	[113]
*Stevia rebaudiana* Bert. (stevia)	SA	CC	SA elicitation, (0.75 mg/L) promoted callus growth rate, callus diameter, and relative callus FW; the addition of 0.25 (mg/L) of SA to the MS medium led to the production of the highest amount of rebaudioside A	[114]
*Artemisia aucheri* Boiss.	SA	ShC	SA pretreatment decreased the effects of PEG-simulated drought stress under in vitro conditions by improving the activity of antioxidant enzymes	[116]
*Echinacea purpurea* L. (echinacea)	SA	ShC	The highest average number of branches formed (33.6 branches/plant part), leaves (33.6 leaves/vegetable part), fresh 1.067 g and dry 0.058 g weights, and a 291.3427 μg/g DW concentration of echinacoside	[117]

Legend: AdvRC—adventitious root culture; CC—callus culture; HR—hairy root culture; ShC—shoot culture; SuspC—suspension culture; TPC—total phenolic content; TFC—total flavonoid content; and AA—antioxidant activity.

#### 2.1.3. Nanoparticles (NP)

Numerous studies show the beneficial effects of nanoparticles in plant tissue culture. These findings focus on the application of NPs to enhance seed germination, plant growth and yield, support genetic modification and plant protection, and improve metabolite content [118]. Utilizing ZnO nanoparticles at concentrations of up to 1 mg/L in MS basal medium resulted in a significantly higher quantity of steviol glycosides in micropropagated shoots of *S. rebaudiana* (Table 3). When ZnO nanoparticles at doses of 100 or 1000 mg/L were added to the MS medium, a negative effect on the phytochemical assays was noticed [119]. There is evidence that 10 mg/L CuO NPs significantly influence shoot organogenesis, steviol glycosides, and the antioxidant activity of in vitro-grown plantlets [120]. The other authors used 2 mg/L of ZnO and 20 mg/L of CuO NPs in an MS nutrient medium and found a positive effect on the in vitro rooting response, content of steviol glycosides, total flavonoid content, total phenolic content, and DPPH free radical scavenging activity of *S. rebaudiana* in vitro shoots [121]. Bimetallic alloys of gold and copper nanoparticles (AuCu NPs) added to the liquid MS medium containing 0.5 mg/L 1-naphthylacetic acid (NAA) have been shown to positively impact biomass and secondary metabolite synthesis in adventitious root cultures of *S. rebaudiana* [122]. Using AuCu NPs at a ratio of 1:3, the largest total phenolic and flavonoid content as well as the maximum DPPH free radical scavenging activity were demonstrated. FeNPs at lower concentrations (45 µg/L) positively impacted morphological growth parameters and steviol glycosides in in vitro cultivated stevia plants; however, FeNPs at higher doses (90 and 135 µg/L) increased total phenolics, total flavonoids, and antioxidant activity. In addition, the higher dose of FeNPs was detrimental to growth characteristics and development [123]. The treatment with a 40 mM concentration showed a significantly higher gene expression of *UGT85C2* (UDP-glycosyltransferases), *KAH* (kaurenoic acid-13 hydroxylase), *UGT74G1*, and *UGT76G1* and a higher glycoside content compared to the control sample [124]. AgNPs have been shown to possess a positive effect on the shoot production and length, as a microbicidal agent, as an inhibitor in ethylene synthesis, in photosynthetic pigment synthesis, nutrient accumulation, antioxidant metabolism, and the ROS generation of *S. rebaudiana* in vitro [125]. Solid culture media have been found to provide a more conducive environment for inducing secondary metabolite biosynthesis in the presence of SiO_2_NPs compared to the liquid medium, possibly due to comparatively slower and reduced nutrient diffusion into the plant cells; however, the liquid medium promotes the morphogenic response in stevia [126]. When Zn-NPs were applied to stevia plants, there was evidence of possible toxicity as shown by morphological data and the generation of ROS-scavenging enzymes under various treatment conditions, but the application of Mg-NPs showed normal plant physiology and stevioside production without any phytotoxic effect [127,128]. Ag-SiO_2_ core-shell nanoparticles induced oxidative stress (H_2_O_2_ production), resulting in lipid peroxidation (increased malondialdehyde accumulation), enhanced activities of catalase, and increased artemisinin content in *A. annua* hairy roots [129]. The study conducted by Bami et al. [130] investigated the impact of four different concentrations of TiO_2_ nanoparticle treatment (0, 10, 20, and 30 mg/L) and NaCl salinity stress (0, 50, 100, and 150 mM NaCl) on the expression of two biosynthetic artemisinin genes, DBR2 and ADS in *A. absinthium*. The findings showed that the treatment with nanoparticles and the stress caused by salinity affected the expressions of these genes significantly. Nanocobalt particles and chitosan nanoparticles have been used (at 0.25, 2.5, and 5 mg/L concentrations) to treat cell suspensions of *A. annua*, and samples are collected after 8, 24, 48, and 72 h [131,132]. The highest artemisinin yield has been obtained 24 h after 5 mg/L nanocobalt treatment, as artemisinin production was 2.25-fold (113.35 mg/g DW) higher than that of the control and 72 h after 5 mg/L nanoparticle treatment. Apart from improved yields of artemisinin, the upregulated expression of *ADS*, *CYP*, *CPR*, *DBR2*, and *ALDH* genes has been reported [131]. The genes *SQS* and *DBR2* had a negative effect on artemisinin accumulation and their expression after the application of cobalt nanoparticles [132]. The treatment of *S. rebaudiana* explants with nanofibers, formed from a derivative of valine as a carrier of the biologically active agent silver atoms (NF-1%Ag and NF-2%Ag) at concentrations of 1, 10, 50, and 100 mg/L, caused hormetic effects on stevia plantlet growth. It has been observed that stimulation of plant growth occurs at low concentrations of 1 to 50 mg/L of nanofibers; however, at a higher dose of 100 mg/L, the inhibition of the values of parameters characterizing plant growth was recorded [133]. The highest amount of stevioside was achieved in plants treated with 100 mg/L NF-1%Ag. In vitro shoots of *S. rebaudiana* treated with nanofibers synthesized by the derivatives of the L-aspartic acid with a dimeric molecular structure (NF2-Ag salt) carrier of silver at varying concentrations (1, 10, 50, 100 mg/L) showed an increase in the soluble sugars, total dicaffeoylquinic (DCQA) acid content, and CQA/DCQA ratio as well as in the micropropagation rate in comparison with control plants in vitro propagated on MS media free of NPs [134].

Similar results for the effect of the nanoparticles on the second metabolites’ synthesis are reported for the plants from the genus *Thymus* L. from the Lamiaceae family [135]. Adding to the MS nutrient medium of 150 mg L^−1^ ZnO NPs played a vital role in enhancing the thymol and carvacrol content in callus cultures of *Thymus* sp. and *Z. multiflora*.

### 2.2. Biotic Elicitors

Biotic elicitors are compounds with a biological origin; they are further classified as microorganism- and polysaccharide-based [25]. An oligosaccharide produced from the cell walls of the endophytic fungus *Colletotrichum* sp. B501 has been examined for its elicitation impact on *A. annua* hairy root cultures at a concentration of 20 mg/L for four days. This resulted in an increase in the yield of 68.29% when compared to the control [136,137] (Table 4). Cerebroside C is a natural glycosphingolipid associated with the fungal plant pathogens *Fusarium oxysporum*, *Pythium* sp., and is reported to be an elicitor for the production of artemisinin using a hairy root culture. In the hairy root culture, exposure to cerebroside caused oxidative bursts that released nitric oxide (NO). This is a key signalling molecule involved in the production of artemisinin [138]. Zheng et al. [139] used a hairy root culture of *A. annua* that was stimulated with a fungal-derived oligosaccharide to investigate the possible function of NO in the production of artemisinin. This resulted in the generation of NO and an enhanced accumulation of artemisinin by a 20-day-old hairy root culture, after four days of exposure to the elicitor. After treating the culture with the oligosaccharide elicitor and sodium nitroprusside, the NO donor, the maximum yield of the metabolite was reported at 28.5 mg/L, which is a two-fold increase over the culture treated with sodium nitroprusside. Hairy roots of *A. annua* treated with a mycelial extract of *Colletotrichum* sp. showed a 44% enhancement of artemisinin accumulation [137]. Calluses and callus-regenerated plantlets have been exposed to endophytic fungi *Penicillium oxalicum* B4 to elicit the cultures for artemisinin accumulation. When exposed to endophytic fungi for 30 days, calluses exhibited no artemisinin production. In contrast, rooted plantlets produced a higher production of artemisinin, estimated at 1.32 mg/g DW, or roughly 43.5% greater than the control [140]. By co-cultivating with the mycorrhiza-like fungus *Piriformospora indica*, *Artemisia annua* shoots have a significant increase in the amount of artemisinin [141]. The biotic elicitors alginate (ALG), casein hydrolysate (CH), PEC, YE, and chitosan have been added to woody plant medium to enhance in vitro shoot growth and biomass accumulation as well as stevioside production, in micropropagated *Stevia rebaudiana*. The biomass accumulation was the highest when 100 µM chitosan was used [142]. The in vitro plantlets treated with either 2.0 g/L YE or 0.5 g/L ALG produced the highest amount of stevioside (14.69 and 14.54 mg/g DW, respectively). In the hairy root cultures of *Artemisia annua* L., chitosan (150 mg/L) significantly enhanced the accumulation of artemisinin [94]. Elshahawy et al. [143] observed that biotic elicitation enhanced the antioxidant capacity, total phenolics, and flavonoid synthesis of *E. purpurea* callus. Using yeast extracts of the fungal elicitors *Aspergillus niger* and *Fusarium oxysporum*, the highest total phenolic content was recorded with 4 g/L YE, 1 g/L *A. niger*, or 0.25 g/L *F. oxysporum*. *Silybum marianum* cell suspension cultures were subjected to seven distinct chitosan concentrations (0.5–50.0 mg/L) [144]. The authors found that chitosan (5.0 mg/L) improved both biomass production and the accumulation of silymarin in *S. marianum* cell suspension cultures. Yeast extract at concentrations of 100 or 200 mg/L effectively promoted the accumulation of santonin and artemisinin in callus cultures of *Artemisia alba* [145]. Some enhancement in artemisinin accumulation was observed in suspension cultures of *A. annua* after the application of yeast extract; however, the addition of chitosan resulted in a reduction in cell growth and artemisinin content [88]. Hairy root cultures of *A. annua* treated with *Piriformospora indica* homogenates and MeJA showed an increase in artemisinin accumulation. The study found that in hairy root cultures of *A. annua*, the effects of *Piriformospora indica* on artemisinin synthesis have been positively correlated with regulatory genes of MVA, MEP, and artemisinin biosynthetic genes *hmgr*, *ads*, *cyp71av1*, *aldh1*, *dxs*, *dxr*, and *dbr2* [95].

## 3. Conclusions and Future Perspectives

Most of the secondary metabolites produced from the Asteraceae plant species are synthesized in low yields in nature. They have various applications in different fields, although their output is limited in terms of attaining commercial production. Current progress in the enhancement of secondary metabolite accumulation includes using elicitors and plant cell tissue and organ cultures as model systems. This review gathers different elicitation strategies for improving the production of key metabolites in Asteraceae plants. The effects of different abiotic and biotic elicitors on secondary metabolite production depend on the specific plant species, duration of elicitor exposure, culture age, and the types of targeted metabolites. In certain cases, combined elicitation appears to be a more successful strategy than single elicitation in terms of secondary metabolite yield. Numerous studies have clarified the role of elicitors in enhancing artemisinin, silymarin, phenolics, caffeic acid derivatives, and steviol glycosides’ production. Research still lacks to promoting the yield of sesquiterpene lactones helenalin and dihydrohelenalin by elicitation in *Arnica montana*, a very old medicinal plant. Although the biosynthetic pathway of parthenolide synthesized by *Tanacetum parthenium* has been elucidated, there is insufficient data on the influence of elicitors applied to the in vitro culture of this valuable species. More studies are needed to increase the production of helenalin and dihydrohelenalin from *A. montana* and parthenolide from the in vitro culture of *T. parthenium*. Using different elicitor treatments including a novel elicitor as nanoparticles is possible to overcome the low yield of biomass and secondary metabolite production as well as to help study analyses of gene expression/transcriptome in the important Asteraceae plants. Elicitation has the potential to be the most significant tool in clarifying the synthesis of desired secondary metabolites in Asteraceae medicinal plants. More future studies are needed to establish elicitor-induced changes that lead to the upregulation of defense-related genes or the downregulation of non-defense-related genes, the transient phosphorylation or dephosphorylation of proteins, and the expression of key enzymes whose information can be used to determine the biosynthetic pathways of Asteraceae secondary metabolites. To gain a better understanding of the regulatory mechanisms controlling secondary metabolism, it is crucial to integrate genomics, transcriptomics, proteomics, and metabolomics. This could lead to the effective metabolic engineering of medicinally essential metabolites in plant in vitro systems.

## Figures and Tables

**Table 3:** Effect of some nanoparticles on biomass accumulation and secondary metabolite content of Asteraceae plants.

Plant Species	Elicitors	Type of In Vitro Culture	Effects	References
	Nanoparticles			
*Stevia rebaudiana* Bert. (stevia)	ZnO	ShC	Higher steviol glycoside content	[119]
*Stevia rebaudiana* Bert. (stevia)	ZnO and CuO	ShC	Higher rooting, steviol glycoside, TPC, TFC, and DPPH free radical scavenging activity	[121]
*Stevia rebaudiana* Bert. (stevia)	CuO	ShC	Positive effect on shoot organogenesis, steviol glycoside content, and AA	[120]
*Stevia rebaudiana* Bert. (stevia)	AuCu	AdvRC	Positive impact on biomass and secondary metabolite production	[122]
*Stevia rebaudiana* Bert. (stevia)	FeNPs	ShC	Improved growth parameters, higher levels of TPC, TFC and steviol glycosides, and AA	[123]
*Stevia rebaudiana* Bert. (stevia)	Ag	ShC	Positive effect on the shoot production and length, as a microbicidal agent, as an inhibitor in ethylene synthesis, in photosynthetic pigment synthesis, nutrient accumulation, AA, and ROS generation	[125]
*Stevia rebaudiana* Bert. (stevia)	SiO_2_	ShC	Enhanced secondary metabolite biosynthesis	[126]
*Stevia rebaudiana* Bert. (stevia)	Zn	ShC	Potential phytotoxicity	[127]
*Stevia rebaudiana* Bert. (stevia)	Mg	ShC	Normal plant physiology and stevioside production	[128]
*Stevia rebaudiana* Bert. (stevia)	Valine derivative nanofibers, carriers of silver atoms (NF-1%Ag and NF-2%Ag)	ShC	The stimulation of plant growth at low concentrations; the highest amount of stevioside at the highest NF-1%Ag concentration	[133]
*Stevia rebaudiana* Bert. (stevia)	L-aspartic acid derivative nanofibers with a dimeric molecular structure, carriers of Ag (NF2-Ag salt)	ShC	Increased soluble sugars and total dicaffeoylquinic (DCQA) acid content, enhanced CQA/DCQA ratio and micropropagation rate	[134]
*Artemisia annua* L. (sweet wormwood)	Ag-SiO_2_	HR	Induced oxidative stress (higher H_2_O_2_ and MDA content), enhanced catalase activity, and increased artemisinin content	[129]
*Artemisia annua* L. (sweet wormwood)	Co	SuspC	Higher artemisinin yield; an upregulated expression of *ADS*, *CYP*, *CPR*, *DBR2*, and *ALDH* genes; a decreased expression of *SQS* and *DBR2* genes	[131,132]

Legend: AdvRC—adventitious root culture; HR—hairy root culture; ShC—shoot culture; SuspC—suspension culture; TPC—total phenolic content; TFC—total flavonoid content; and AA—antioxidant activity.

**Table 4:** Effect of biotic elicitors on biomass accumulation and secondary metabolite content in in vitro culture of Asteraceae plants.

Biotic Elicitor				
Plant Species	Biotic Elicitors	Type of In Vitro Culture	Effects	References
*Artemisia annua* L. (sweet wormwood)	Cell wall’s oligosaccharide from *Colletotrichum* sp. B501	HR	Increased artemisinin yield by 68.29%	[136]
*Artemisia annua* L. (sweet wormwood)	Cerebroside from fungal source	HR	Oxidative bursts that released nitric oxide (NO); increased artemisinin yield 2.3 folds	[138]
*Artemisia annua* L. (sweet wormwood)	Oligosaccharide from *Fusarium oxysporum* mycelium	HR	Increased artemisinin content from 0.7 mg/g DW to 1.3 mg/g DW	[139]
*Artemisia annua* L. (sweet wormwood)	*Penicillium oxalicum* B4	In vitro-grown rooted plantlets	Increased artemisinin content by 43.5%	[140]
*Stevia rebaudiana* Bert. (stevia)	Alginate, casein hydrolysate, pectin, yeast extract, and chitosan	ShC	Biomass accumulation is the highest when 100 µM chitosan is used. The in vitro plantlets treated with either 2.0 g/L YE or 0.5 g/L ALG produced the highest amount of stevioside (14.69 and 14.54 mg/g DW, respectively).	[142]
*Artemisia annua* L. (sweet wormwood)	Chitosan (150 mg/L)	HR	Enhanced the accumulation of artemisinin	[94]
*Echinacea purpurea* L. (echinacea)	Fungal elicitors yeast extract, *Aspergillus niger* and *Fusarium oxysporum*	CC	Total phenolics is recorded with 4 g/L YE, 1 g/L A. niger or 0.25 g/L *F. Oxysporum*	[143]
Silybum *marianum* L. (milk thistle)	Chitosan (0.5–50.0 mg/L)	SuspC	Chitosan (5.0 mg/L) improved both biomass production and the accumulation of silymarin	[144]
*Artemisia alba* Turra. (white wormwood)	Yeast extract at a concentration of 100 or 200 mg/L	CC	An enhanced accumulation of santonin and artemisinin	[145]
*Artemisia annua* L. (sweet wormwood)	Yeast extract Chitosan	SuspC	Increased artemisinin accumulation	[88]
*Artemisia annua* L. (sweet wormwood)	*Piriformospora indica* homogenates and MeJA	HR	An increase in artemisinin accumulation; the effects of *Piriformospora indica* on artemisinin synthesis, positively correlated with regulatory genes of MVA, MEP, and artemisinin biosynthetic genes *hmgr*, *ads*, *cyp71av1*, *aldh1*, *dxs*, *dxr*, and *dbr2*	[95]

Legend: CC—callus culture; HR—hairy root culture; ShC—shoot culture; SuspC—suspension culture.

## Data Availability

Not applicable.
